# Supplementary material for: Distinct iron cycling in a Southern Ocean eddy
Source: Nat Commun. 2020 Feb 11;11:825. doi: 10.1038/s41467-020-14464-0 (PMC7012851; doi:10.1038/s41467-020-14464-0)
Supplement: Supplementary file 1 — Supplementary Information [file 41467_2020_14464_MOESM1_ESM.pdf]

Supplementary Information for

Distinct iron cycling in a Southern Ocean eddy

by Ellwood et al.

Supplementary Methods

Extended 1D Model Description

The 1D model aimed to simulate the interactions between phytoplankton group with reference to key nutrients, including nitrate, phosphate and iron (Supplementary Figure 7). The model is largely based on the one developed by *Schlosser et al.* [1].

In the model, phytoplankton production is expressed as a function of nitrogen biomass. The limiting term for phytoplankton production is expressed as:

$$\gamma_{\text{phyto}} = \min\left[\frac{[N]}{k_{\text{phyto.N}}+[N]}, \frac{[P]}{k_{\text{phyto.P}}+[P]}, \frac{PAR}{k_{\text{phyto.PAR}}+PAR}, \frac{[Fe]}{k_{\text{phyto.Fe}}+[Fe]}\right] \quad 1$$

The definitions for each symbol and their values are presented in Supplementary Table 1.

In the upper water column, at and above 100 m (i.e. the deep chlorophyll maximum), the regeneration of nutrients back into solution is expressed as a linear function relative to biomass. Below 100 m, the rate of nutrient regeneration decreases following the Martin POC attenuation expression power law [2] :

$$k_{\text{regen.z}} = k_{\text{regen.z}_0}\,(z/z_0)^b \quad 2$$

This function effectively trends the sinking particles as transitioning from fresh to refractory with depth. For simplicity, the model used single values of k_{regen} (0.35 d^{-1}) and the power-law exponent b (-0.858) [2] for all components. For Fe a single value of 0.03 d^{-1} was used for $k_{regen,}$ which was also attenuated using Supplementary Equation 2.

For dissolved inorganic nitrogen (NO_3), the following Supplementary Equations 3 and 4 express its uptake by phytoplankton, its exchange between boxes and its regeneration from phytoplankton, expressed here as particulate organic nitrogen (PON):

$$\frac{\mathrm{d}[NO_3]_{\mathrm{j-1}}}{\mathrm{dt}} = k_{\text{mix}}\cdot\left([NO_3]_{\mathrm{j}} + [NO_3]_{\mathrm{j-2}}\right) - 2\cdot k_{\text{mix}}\cdot[NO_3]_{\mathrm{j-1}} + k_{\text{regen}}\cdot[PON]_{\mathrm{j-1}} - \gamma_{\text{phyto}}\cdot \mu_{\text{max.phyto}}\cdot[NO_3]_{\mathrm{j-1}} \quad 3$$

Note that the maximum uptake rate $\mu_{max\cdot phyto}$ is in units of nitrogen.

Overall, the change in PON can be expressed as:

$$\frac{\mathrm{d}[PON]_{\mathrm{j-1}}}{\mathrm{dt}} = \gamma_{\text{phyto}}\cdot\mu_{\text{max.phyto}}\cdot[NO_3]_{\mathrm{j-1}} + k_{\text{sink}}\cdot[PON]_{\mathrm{j}} - k_{\text{sink}}\cdot[PON]_{\mathrm{j-1}} + k_{\text{regen}}\cdot[PON]_{\mathrm{j-1}} \quad 4$$

In this expression (Supplementary Equation 4), and for the other particle expressions (Supplementary Equations 6 and 11), the mixing terms do not appear, because the sinking rate of particles (at 10 m d^{-1}) overwhelms the mixing (range 1.2 to 6 m d^{-1}) contribution to their distributions, especially during summertime.

For dissolved inorganic phosphate (PO_4), the following Supplementary Equation expresses its uptake by phytoplankton to form particulate organic phosphorus (POP), its exchange between boxes and its regeneration from phytoplankton:

$$\frac{\mathrm{d}[PO_4]_{\mathrm{j-1}}}{\mathrm{dt}} = k_{\mathrm{mix}} \cdot \left([PO_4]_{\mathrm{j}} + [PO_4]_{\mathrm{j-2}}\right) - 2 \cdot k_{\mathrm{mix}} . [PO_4]_{\mathrm{j-1}} + k_{\mathrm{regen}} \cdot [\mathrm{POP}]_{\mathrm{j-1}} - \gamma_{\mathrm{phyto}} \cdot \mu_{\mathrm{max.phyto}} \cdot R_{\mathrm{N.P.phyto}} [PO_4]_{\mathrm{j-1}} \quad 5$$

$$\frac{\mathrm{d}[POP]_{\mathrm{j-1}}}{\mathrm{dt}} = \gamma_{\mathrm{phyto}} \cdot \mu_{\mathrm{max.phyto}} \cdot R_{\mathrm{NP.phyto}} \cdot [PO_4]_{\mathrm{j-1}} + k_{\mathrm{sink}} \cdot [\mathrm{POP}]_{\mathrm{j}} - k_{\mathrm{sink}} \cdot [POP]_{\mathrm{j-1}} - k_{\mathrm{regen}} \cdot [POP]_{\mathrm{j-1}} \quad 6$$

For dFe, the following Supplementary Equation expresses its uptake by phytoplankton, its exchange between boxes and its regeneration from sinking particulate matter:

$$\frac{\mathrm{d}[\mathrm{Fe_{diss}}]_{\mathrm{j\text{-}1}}}{\mathrm{dt}} = \mathrm{k_{mix}} \cdot \left([\mathrm{dFe}]_{\mathrm{j}} + [\mathrm{dFe}]_{\mathrm{j\text{-}2}}\right) \text{-} 2 \cdot \mathrm{k_{mix}} \cdot [\mathrm{dFe}]_{\mathrm{j\text{-}1}} + \mathrm{k_{regen.Fe}} \cdot [\mathrm{Fe_{part}}]_{\mathrm{j\text{-}1}} - \gamma_{\mathrm{phyto}} \cdot \mu_{\mathrm{max.phyto}} \cdot \mathrm{R_{N.Fe.phyto}} \cdot \left[\mathrm{Fe'}\right]_{\mathrm{j\text{-}1}} \quad 7$$

where Fe′ represents inorganic Fe. The partitioning between organically complexed (FeL) and uncomplexed Fe, i.e. FeL vs Fe^{III} or Fe′, was simulated using the following Supplementary Equation [3]:

$$[Fe^{III}]/[FeL] = [Fe^{III}]/C_L + 1/(C_L K'_{FeL}) \quad 8$$

Noting that

$$[Fe^{III}] \quad = [Fe']/\alpha_{Fe} \quad 9$$

where α_{Fe} represents the coefficient for inorganic complexation of Fe^{III} in seawater (10^{10}) [ref 4,5]

Supplementary Equation 8 was rearranged to give:

$$[Fe^{III}]^2 \cdot \alpha_{\mathrm{Fe}} \cdot K'_{\mathrm{FeL}} + [Fe^{III}] \cdot (\alpha_{\mathrm{Fe}} + K'_{\mathrm{FeL}} \cdot C_{\mathrm{L}} - C_{\mathrm{Fe}} \cdot K'_{\mathrm{FeL}}) - C_{\mathrm{Fe}} = 0 \quad 10$$

where C_{Fe} represents the dFe concentration, K'_{FeL} presents the conditional stability constant for the Fe^{III}-ligand complex, C_L presents the total concentration of Fe-binding ligands in solution. Here K'_{FeL}, C_L and α_{Fe} were set to 10^{20}, 0.6 nmol L^{-1} and 10^{10}, respectively [3-6]. Supplementary Equation 10 was solved within each iterative model cycle.

The overall change in particulate Fe concentration can be expressed as:

$$\frac{\mathrm{d}[Fe_{\mathrm{part}}]_{\mathrm{j-1}}}{\mathrm{dt}} = \gamma_{\mathrm{phyto}} \cdot \mu_{\mathrm{max.phyto}} \cdot R_{\mathrm{N.Fe.phyto}} \cdot [Fe']_{\mathrm{j-1}} + k_{\mathrm{sink}} \cdot [Fe_{\mathrm{part}}]_{\mathrm{j}} - k_{\mathrm{sink}} \cdot [Fe_{\mathrm{part}}]_{\mathrm{j-1}} - k_{\mathrm{regen.Fe}} \cdot [Fe_{\mathrm{part}}]_{\mathrm{j-1}} \quad 11$$

For the deepest model box, box *j-n*, nitrate, phosphate and dFe were supplied into this box from below. These inputs were required to balance the loss of particulate organic nitrogen, particulate organic phosphate and particulate Fe associated with sinking matter that sinks out of this box. Particulate Fe was also supplied in the shallowest model box, box *j*, through atmospheric dust deposition.

For model runs we simulated dissolved nitrate, phosphate and Fe profiles to a depth of 500 m with depth intervals of 20 m. Seasonality was introduced into the model by varying the light (PAR) and mixing component using a cosine function, such that the light field was highest at the summer solstice (22 December) and mixing was highest at the winter solstice (21 June), (Supplementary Figure 8) [ref 7]. Data were extracted from the model for 1 April, which is close to the time the cold core eddy was sampled.

The model was run to an annual steady-state with respect to the deeper ocean (>250 m) and the model was 'calibrated' to reference profiles of nitrate and dFe from within the cold core eddy. This was achieved by adjusting the supply of nutrients into the deepest box, box *j-n* (Supplementary Table 3, Supplementary Figure 7). Note that there was a change in water mass below 280 m. Hence, there is a large discrepancy between the observed dFe values below this depth and our model Fe results below 280 m (Supplementary Figure 8).

Iron isotope fractionation was simulated in the model by applying fractionation factors (α) to the following processes: Fe uptake by phytoplankton, Fe regeneration from organic matter, Fe′ scavenging from solution and Fe complexation by natural organic ligands (Supplementary Table 2). For each of these processes, isotope fractionation is in the expected direction for kinetic fractionation, i.e. ^{54}Fe reacts faster than ^{56}Fe. Thus, during Fe uptake by phytoplankton, the resulting biomass will have an isotopically lighter composition than the dFe substrate, which, in turn, will become progressively heavier. Regeneration preferentially releases ^{54}Fe, lightening the dFe isotopic pool and rendering the remnant particle pool isotopically heavier. Understanding the influence of the ligand complexation process on the dissolved and particulate Fe isotopic compositions is dependent on the operational definition of 'dissolved'. As measured here, the operational separation of particles and dissolved Fe is by filtration at a 0.2 µm pore size such that the dFe measurement is the sum of the inorganic, colloidal and ligand-bound Fe. The ligand-bound Fe

component dominates the total dFe pool, especially in surface waters. Thus, Fe released from ligands and taken-up by phytoplankton is lighter than that of the operationally measured dFe [8], which in turn leads to a lighter particulate Fe isotopic composition that would otherwise arise without the influence of ligand complexation. Scavenging of Fe' from solution is expected to result in ^{54}Fe enrichment of the particulate phase leading to an isotopically light particulate pool relative to the dFe pool.

As mentioned, atmospheric Fe is supplied into the particulate phase for the top box *j*. At the bottom, particles are allowed to exit the *j*-*n* box and this is balanced by an upward supply flux of dfe and nutrients into this box. The isotopic composition of the upward supply dFe into the *j*-*n* box is set to 0.1 ‰, and atmospheric supply of Fe into the *j* box was also set to 0.1 ‰.

Supplementary Discussion

Low atmospheric input

In-eddy particulate and dissolved aluminium concentrations were low (range: 0.08 - 0.27 nmol kg^{-1}) between 15 and 200 m (Supplementary Figures 2 and 5). For the SAZ station, particulate and dissolved aluminium concentration were also low (range: 0.09 – 0.15 nmol kg^{-1}) between 15 and 200 m (Supplementary Figure 2 and 5). The low surface ocean particulate aluminium concentration for these two stations is consistent with low atmospheric dust deposition for the region and suggests that the elevated Fe isotope composition observed for the euphotic zone for the cold core eddy is not a result of atmospheric deposition of Fe with heavy isotope composition. Indeed, the isotope composition of crustal Fe is typically around 0.1 ‰ [ref 9,10], which is well below measured values within the eddy.

Iron isotope fractionation observations vs model

The changes in $\delta^{56}Fe_{dissolved}$ between 15 and 100 m, resulting in an apparent ε of -2.3 ‰, could be a consequence of several processes including 1) complexation to natural organic ligands (Supplementary Table 2) [ref 8], 2) biological fractionation within the euphotic zone and, 3) isotope fractionation upon Fe regeneration from organic matter and 4) the scavenging of Fe from solution[11]. We further explored the possible impact of these processes in the 1-D model by running it over a yearly cycle to mimic the seasonal conversion of dFe to pFe in the euphotic zone followed by the remineralization of pFe at depth (Supplementary Figure 8).

Dissolved and particulate Fe isotope fractionation with the cold core eddy could be best simulated using a combination of isotope fractionation processes including pFe regeneration, dFe scavenging from solution and dFe complexation to natural organic ligands or with a single fractionation factor of α_{uptake} of 0.999 (ε = -1‰) associated with dFe uptake by cells (Supplementary Figure 9). For these various processes, we applied fractionation factors from the literature (Supplementary Table 2). When isotope fractionation associated with organic ligand complexation, Fe regeneration from sinking organic matter and dFe scavenging are considered with fractionation with dFe uptake, the overall result is the same as when only a single isotope process is considered with dFe uptake by phytoplankton (Figure 4 and Supplementary Figure 9). We also found that applying a fractionation factor equivalent to ε_{uptake} equal to -2.3 ‰ (α_{uptake}) could not match the measured dFe isotope profile (Figure 4). Note that these fits are only relevant across the euphotic zone to a depth of 280 m; below this depth, there was a change in water mass.

Supplementary Tables

Supplementary Table 1 Intracellular (titanium washed) size fractioned carbon uptake results for profiles Cold Core Eddy station, the Subantarctic Zone station and at the Southern Ocean Time Series station.

Depth (m)	% Incident irradiance	>20 μm (mmol m^{-3} d^{-1})		2-20 μm (mmol m^{-3} d^{-1})		0.2 - 2 μm (mmol m^{-3} d^{-1})		Sum (mmol m^{-3} d^{-1})	
Subantarctic Zone station (-51.46°S; 148.52°E)									
15	80	0.056	± 0.006	0.118	± 0.011	0.134	± 0.015	0.308	± 0.014
30	50	0.053	± 0.001	0.107	± 0.027	0.114	± 0.018	0.273	± 0.023
40	20	0.039	± 0.008	0.090	± 0.007	0.122	± 0.002	0.251	± 0.008
55	10	0.034	± 0.003	0.079	± 0.000	0.104	± 0.019	0.217	± 0.013
85	5	0.013	± 0.000	0.031	± 0.006	0.035	± 0.004	0.079	± 0.005
100	1	0.008	± 0.001	0.011	± 0.002	0.019	± 0.004	0.038	± 0.004
Cold Core Eddy (-50.39°S; 147.08°E)									
15	80	0.040	± 0.010	0.046	± 0.028	0.061	± 0.003	0.147	± 0.021
30	50	0.036	± 0.002	0.041	± 0.006	0.049	± 0.004	0.125	± 0.005
40	20	0.022	± 0.003	0.025	± 0.008	0.028	± 0.000	0.075	± 0.006
55	10	0.016	± 0.002	0.021	± 0.003	0.031	± 0.001	0.067	± 0.002
85	5	0.007	± 0.001	0.008	± 0.000	0.013	± 0.001	0.028	± 0.001
100	1	0.005	± 0.000	0.005	± 0.000	0.008	± 0.001	0.018	± 0.000
Southern Ocean Time Series station (-46.77°S; 142.03°E)									
15	80	0.040	± 0.000	0.100	± 0.010	0.240	± 0.030	0.380	± 0.020
30	50	0.050	± 0.010	0.100	± 0.000	0.340	± 0.050	0.490	± 0.040
40	20	0.070	± 0.020	0.100	± 0.000	0.300	± 0.040	0.460	± 0.030
55	10	0.050	± 0.000	0.090	± 0.000	0.300	± 0.020	0.430	± 0.010
85	5	0.020	± 0.000	0.040	± 0.000	0.140	± 0.000	0.190	± 0.000
100	1	0.010	± 0.000	0.020	± 0.000	0.050	± 0.000	0.080	± 0.000

Supplementary Table 2. Dissolved iron concentrations and isotope values along supporting nutrient data samples collected in 2016 at the Cold Core Eddy station, the Subantarctic Zone station and at the Southern Ocean Time Series station.

Depth (db)	Fe (nmol kg^{-1})	$\delta^{56}Fe_{IRMM014}$ (‰)$	NO_3 (μmol L^{-1})	PO_4 (μmol L^{-1})	Si (μmol L^{-1})	NH_4 (μmol L^{-1})	NO_2 (μmol L^{-1})
Cold Core Eddy (-50.39°S; 147.08°E)#							
15	0.024	1.18 ± 0.51	22.2	1.56	3.3	0.32	0.28
40	0.022	1.28 ± 0.39	22.2	1.56	3.3	0.32	0.28
70	0.018	1.10 ± 0.43	22.4	1.58	3.4	0.38	0.28
100	0.028	0.42 ± 0.64	23.3	1.67	3.6	0.81	0.30
150	0.027	0.00 ± 0.32	24.4	1.74	6.5	0.59	0.55
200	0.033	0.21 ± 0.40	25.7	1.79	9.7	0.21	0.07
300	0.41	0.64 ± 0.05	26.6	1.84	14.2	0.15	0.01
750	0.71	0.18 ± 0.04	33.2	2.31	41.6	0.09	0.01
1000	0.51	0.06 ± 0.05	34.7	2.42	58.2	0.09	0.01
1250	0.52	0.20 ± 0.06	34.8	2.42	68.8	0.06	0.01
1500	0.37	-0.10 ± 0.06	33.9	2.36	76.0	0.03	0.01
Subantarctic Zone station (-51.46°S; 148.52°E)							
15	0.060	0.35 ± 0.17	14.5	1.11	1.3	0.24	0.28
40	0.036	-0.06 ± 0.29	14.5	1.11	1.3	0.23	0.28
70	0.047	0.36 ± 0.26	14.5	1.11	1.3	0.27	0.28
100	0.035	-0.13 ± 0.26	14.4	1.10	1.4	0.23	0.28
150	0.046	0.13 ± 0.22	15.7	1.17	3.0	0.10	0.07
200	0.068	0.04 ± 0.20	15.6	1.15	3.9	0.14	0.01
300	0.087	0.02 ± 0.13	16.4	1.21	4.4	0.12	0.01
500	0.13	-0.23 ± 0.09	18.6	1.35	5.7	0.14	0.01
750	0.30	-0.15 ± 0.06	26.2	1.83	16.0	0.13	0.00
1000	0.35	0.10 ± 0.05	31.0	2.17	30.5	0.12	0.00
1250	0.41	0.00 ± 0.06	33.8	2.38	49.6	0.12	0.00
1500	0.44	-0.06 ± 0.06	34.9	2.46	66.0	0.08	0.00
Southern Ocean Time Series station (-46.77°S; 142.03°E)							
15	0.063	0.34 ± 0.14	4.5	0.46	1.3	0.19	0.17
40	0.057	0.30 ± 0.21	4.6	0.46	1.3	0.17	0.17
70	0.064	0.28 ± 0.14	4.9	0.48	1.3	0.19	0.18
100	0.21	0.60 ± 0.07	10.7	0.81	3.5	0.02	0.03
150	0.19	0.36 ± 0.10	12.3	0.91	4.1	0.00	0.01
200	0.21	0.24 ± 0.07	13.5	0.98	4.6	0.03	0.01
300	0.19	0.41 ± 0.09	14.9	1.08	5.1	0.04	0.01
500	0.22	-0.11 ± 0.07	16.7	1.20	5.8	0.05	0.02
750	0.50	0.18 ± 0.04	23.5	1.62	13.6	0.02	0.00
1000	0.57	0.44 ± 0.04	29.0	2.00	28.5	0.02	0.01
1250	0.53	0.30 ± 0.05	33.3	2.30	48.6	0.03	0.00
1500	0.56	0.22 ± 0.04	35.0	2.43	70.6	0.01	0.00

data from the 500 m depth were not include as the collection bottle misfired at the wrong depth

$ errors represent 2 s.e.m. for each measurement

Supplementary Table 3. Particulate iron, aluminium and phosphorus concentration and isotope data for samples collected in 2016 at the Cold Core Eddy station and the Subantarctic Zone station.

Depth (m)	Fe (nmol L^{-1})	$\delta^{56}Fe_{IRMM014}$ (‰)$^{\$}$	Al (nmol L^{-1})	P (nmol L^{-1})
Cold Core Eddy station (-50.39°S; 147.08°E)				
Profile 1				
40	0.023	0.33 ± 0.08	0.06	13
90	0.056	0.32 ± 0.07	0.15	19.5
150	0.022	0.16 ± 0.03	0.03	2.9
250	0.056	0.12 ± 0.02	0.10	2.4
500	0.137	-0.05 ± 0.02	0.70	2.1
Profile 2				
40	0.023	0.31 ± 0.06	0.07	14.1
90	0.020	0.21 ± 0.06	0.04	7.1
150	0.025	0.55 ± 0.05	0.05	3.7
250	0.047	-0.05 ± 0.06	0.07	2.2
500	0.067	0.00 ± 0.06	0.12	1.5
Subantarctic station (-51.46°S; 148.52°E)				
40	0.024	0.15 ± 0.05	0.07	11.4
90	0.054	0.07 ± 0.05	0.20	14
150	0.016	0.04 ± 0.05	0.04	0.1
250	0.040	0.02 ± 0.05	0.10	1.8
500	0.116	-0.06 ± 0.06	0.30	2.8

$^{\$}$ errors represent 2 s.e.m. for each measurement

Supplementary Table 4. Intracellular (titanium washed) size fractioned iron uptake results for profiles Cold Core Eddy station, the Subantarctic Zone station and at the Southern Ocean Time Series station.

Depth (m)	% Incident irradiance	>20 μm (nmol m^{-3} d^{-1})		2-20 μm (nmol m^{-3} d^{-1})		0.2 - 2 μm (nmol m^{-3} d^{-1})		Sum (nmol m^{-3} d^{-1})	
Subantarctic Zone station (-51.46°S; 148.52°E)									
15	80	1.64	± 0.33	2.74	± 0.33	6.61	± 0.57	11.0	± 0.5
30	50	1.72	± 0.21	3.91	± 1.53	8.35	± 0.68	14.0	± 1.2
40	20	1.46	± 0.01	2.74	± 0.83	5.65	± 2.78	9.86	± 2.1
55	10	1.56	± 0.45	3.28	± 0.74	8.27	± 0.22	13.1	± 0.6
85	5	1.43	± 0.07	3.61	± 0.14	7.94	± 0.12	13.0	± 0.1
100	1	1.10	± 0.08	2.94	± 0.46	5.72	± 0.21	9.8	± 0.4
Cold Core Eddy (-50.39°S; 147.08°E)									
15	80	4.86	± 1.25	4.61	± 3.02	17.3	± 5.58	26.8	± 4.6
30	50	5.07	± 0.71	5.22	± 0.45	19.0	± 0.97	29.3	± 0.9
40	20	4.60	± 0.26	4.09	± 0.76	10.5	± 8.23	19.2	± 5.9
55	10	3.23	± 0.27	3.48	± 0.03	12.8	± 1.41	19.5	± 1.0
85	5	2.01	± 0.46	2.75	± 0.23	10.7	± 1.73	15.5	± 1.3
100	1	1.86	± 0.28	2.91	± 0.86	6.3	± 2.3	11.1	± 1.7
Southern Ocean Time Series station (-46.77°S; 142.03°E)									
15	80	0.97	± 0.20	3.02	± 0.40	16.3	± 0.5	20.3	± 0.4
30	50	1.10	± 0.10	2.64	± 0.10	27.6	± 0.1	31.3	± 0.2
40	20	1.31	± 0.00	2.82	± 0.40	26.8	± 1.9	30.9	± 1.4
55	10	0.89	± 0.30	3.00	± 0.30	23.3	± 7.9	27.7	± 5.6
85	5	0.76	± 0.30	3.50	± 0.40	23.4	± 5.8	27.7	± 4.1
100	1	1.13	± 0.10	3.23	± 0.10	23.0	± 7.5	27.4	± 5.3

Supplementary Table 5. Input parameters for 1D model used to generate profiles of dissolved Fe along with references to certain parameters used to constrain the model

Symbol	Description	Units	Value	Reference/notes
PON	Phytoplankton biomass expressed as PON	μM (N)		
N	Dissolved inorganic nitrate (DIN)	μM		
P	Dissolved inorganic phosphate (DIP)	μM		
dFe	Dissolved Fe	nM		
POP	Particulate organic phosphorus	μM		
PFe	Particulate Fe	nM		
PAR	Photosynthetic Active Radiation	μmol (photons) m^{-2} s^{-1}	10	This represents the average summertime value between 0 and 20 m for the first box. PAR was adjusted to simulate as a seasonal light cycle.
Dust	Atmospheric Dust input	g m^{-2} d^{-1}	2 $x10^{-5}$	This represents a Southern Ocean input value [12]. We assumed that the dust is 5% w/w Fe and has a solubility of 3%.
$\mu_{max.phyto}$	Maximum overall phytoplankton growth rate	d^{-1}	0.45	Based on the growth rate for Southern Ocean phytoplankton [13]
$K_{phyto.N}$	Phytoplankton biomass DIN half-saturation constant	μM	0.025	[14,15]
$K_{phyto.P}$	Phytoplankton biomass DIP half-saturation constant	μM	0.01	[16]
$K_{phyto.dFe}$	Phytoplankton biomass dFe half-saturation constant	nM	0.05	[17]
$K_{phyto.PAR}$	Phytoplankton biomass PAR half-saturation constant	μmol (photons) m^{-2} s^{-1}	10	[18]
R.N.P.phyto	Phytoplankton N:P uptake ratio	mol:mol	16	[19]
$R_{Fe.N.phyto}$	Uptake ratio for uncomplexed inorganic Fe relative to nitrate by phytoplankton	μmol:mol	27	Based on Fe:C uptake ratio of 182 μmol:mol for the CCE at 15 m.

k_{mix}	water exchange rate between boxes	$m\ d^{-1}$	1-6	Variable. Mixing was adjusted to simulate a seasonal mixing cycle
k_{sink}	sinking rate for particulate matter	$m\ d^{-1}$	10	
$k_{scav.Fe}$	Scavenging rate for removal of dFe	d^{-1}	$75\ x10^{-5}$	Value is taken from Moore and Braucher [20] for scavenging at the base of the euphotic zone ~ 100 m.
$k_{regen.Fe}$	Regeneration rate for particulate Fe	d^{-1}	0.03	This represents the refractory nature of Fe
k_{regen}	Regeneration rate for particulate nitrogen and phosphorus	d^{-1}	0.35¶	
N_{j-n}	DIN input flux into box j-n	$\mu mol\ m^{-2}\ d^{-1}$	0.0028	
Fe.N.DW	Deep water dFe:NO_3 input flux supply ratio	mmol:mol	0.01	Base on the deep ratio dFe:NO_3
N.P.DW	Deep water N:P input flux supply ratio	mol:mol	16	

¶ This rate was held constant to the depth at which the peak biomass occurred, below which the rate of regeneration declined following the Martin POC regeneration expression (Supplementary Equation 2).

Supplementary Table 6. Iron isotope fractionation factors relative to the dissolved phase.

Description	Values	Reference/notes
α_{uptake}	0.9995-0.9977	Adjusted to fit dFe and pFe profiles
$\alpha_{complexation}$	1.0006	Dideriksen et al. [8]
$\alpha_{scavenging}$	0.9997	John and Adkins [11]
$\alpha_{regeneration}$	0.99985	Adjusted to fit profiles

Supplementary Figures

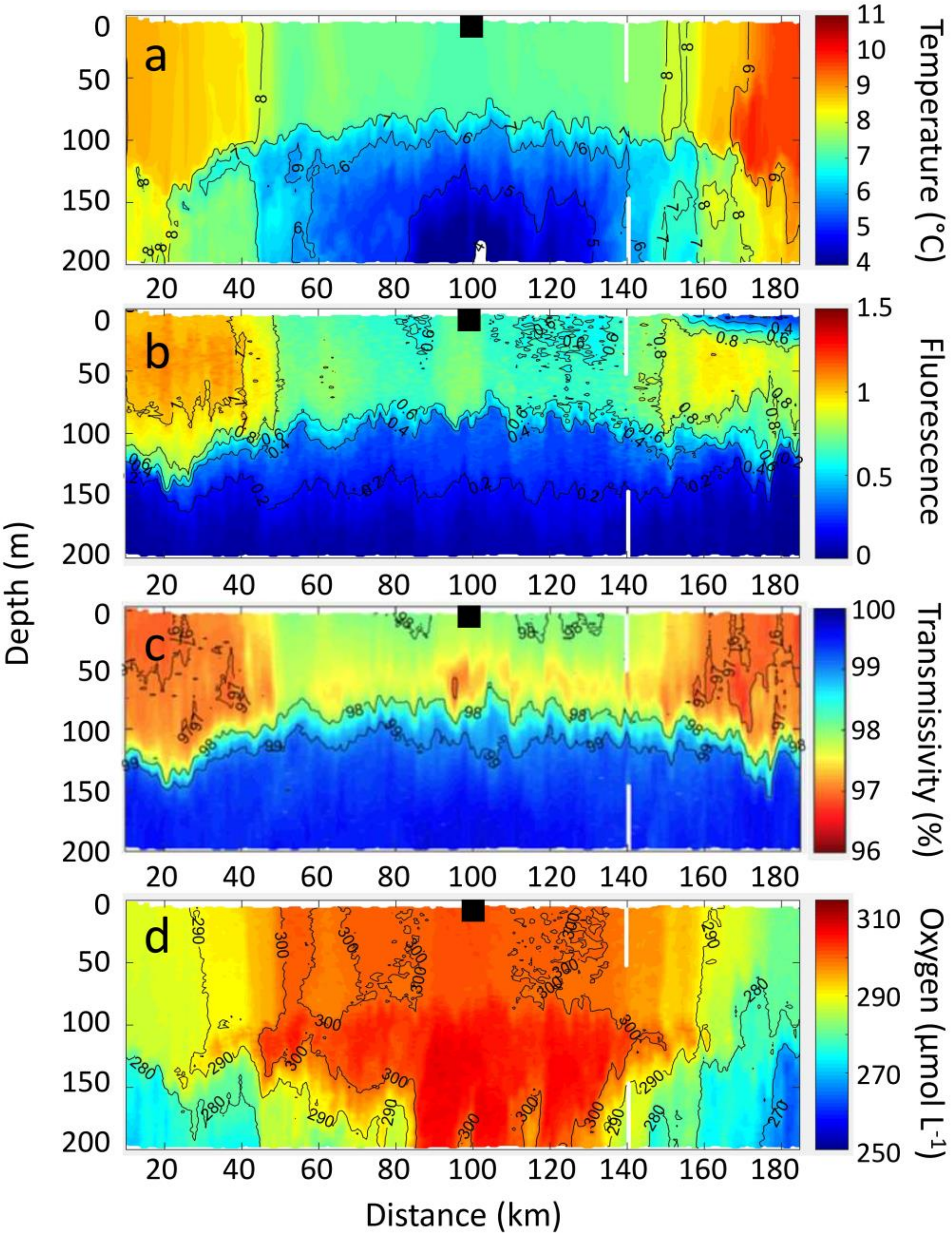

Supplementary Figure 1. Upper ocean physics and chemistry across the eddy. Sections (0 – 200 m) of **a.** temperature, **b.** fluorescence (a proxy for chlorophyll *a* concentration), **c.** transmissivity and **d.** oxygen concentration versus transect distance. The cold core eddy Triaxus transect runs from 50.38°S; 145.66°E to 50.37°S. See Figure 1 for a map of the region. The black square indicates the location of the sample collection station within the cold core eddy.

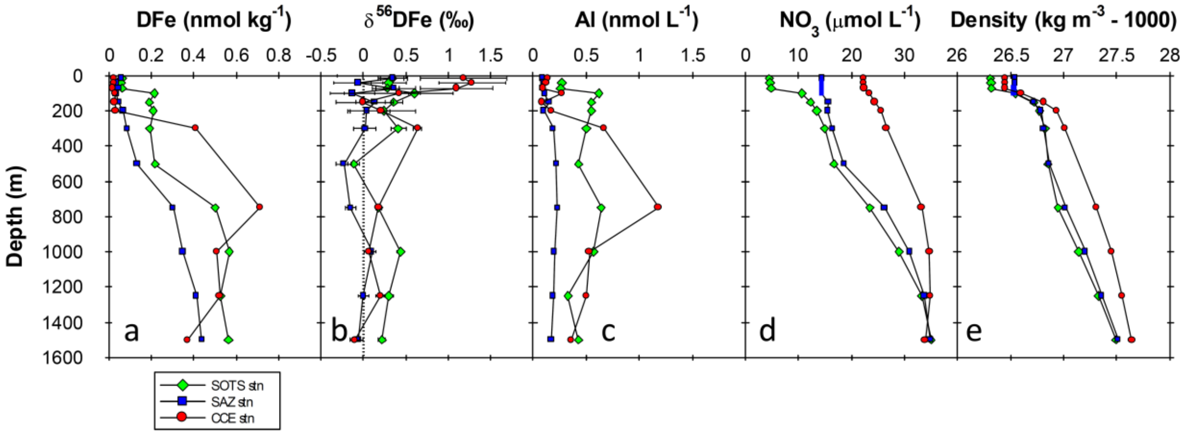

Supplementary Figure 2. Metal, nutrient and density depth profiles. Depth profiles of **a.** dissolved Fe concentration, **b.** isotope composition of dissolved Fe, **c.** dissolved aluminium concentration, **d.** nitrate concentration and **e.** density for profiles collected from the CCE, SAZ and SOTS sites. Error bars for isotope measurements represent 2 s.e.m..

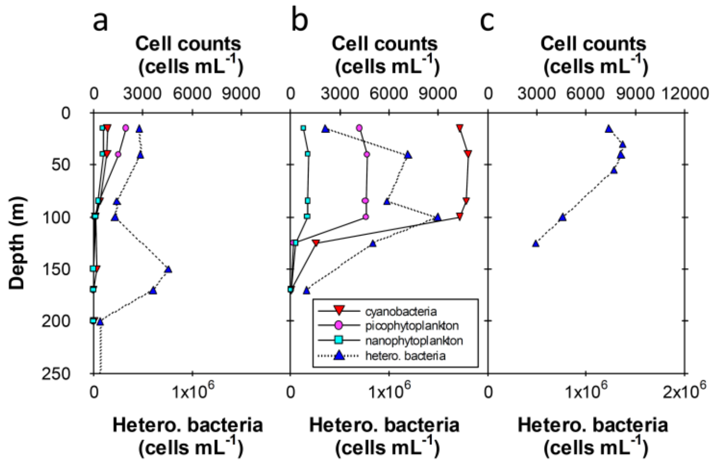

Supplementary Figure 3. Phytoplankton Cell counts. Cell counts for cyanobacteria, pico and nano eukaryotic phytoplankton and heterotrophic bacteria collected at **a.** the Cold Core eddy station (CCE), **b.** the Subantarctic zone station (SAZ) and **c.** Southern Ocean Time Station (SOTS). Note the differenced scale for the heterotrophic bacteria cell counts between panels **b** and **c**.

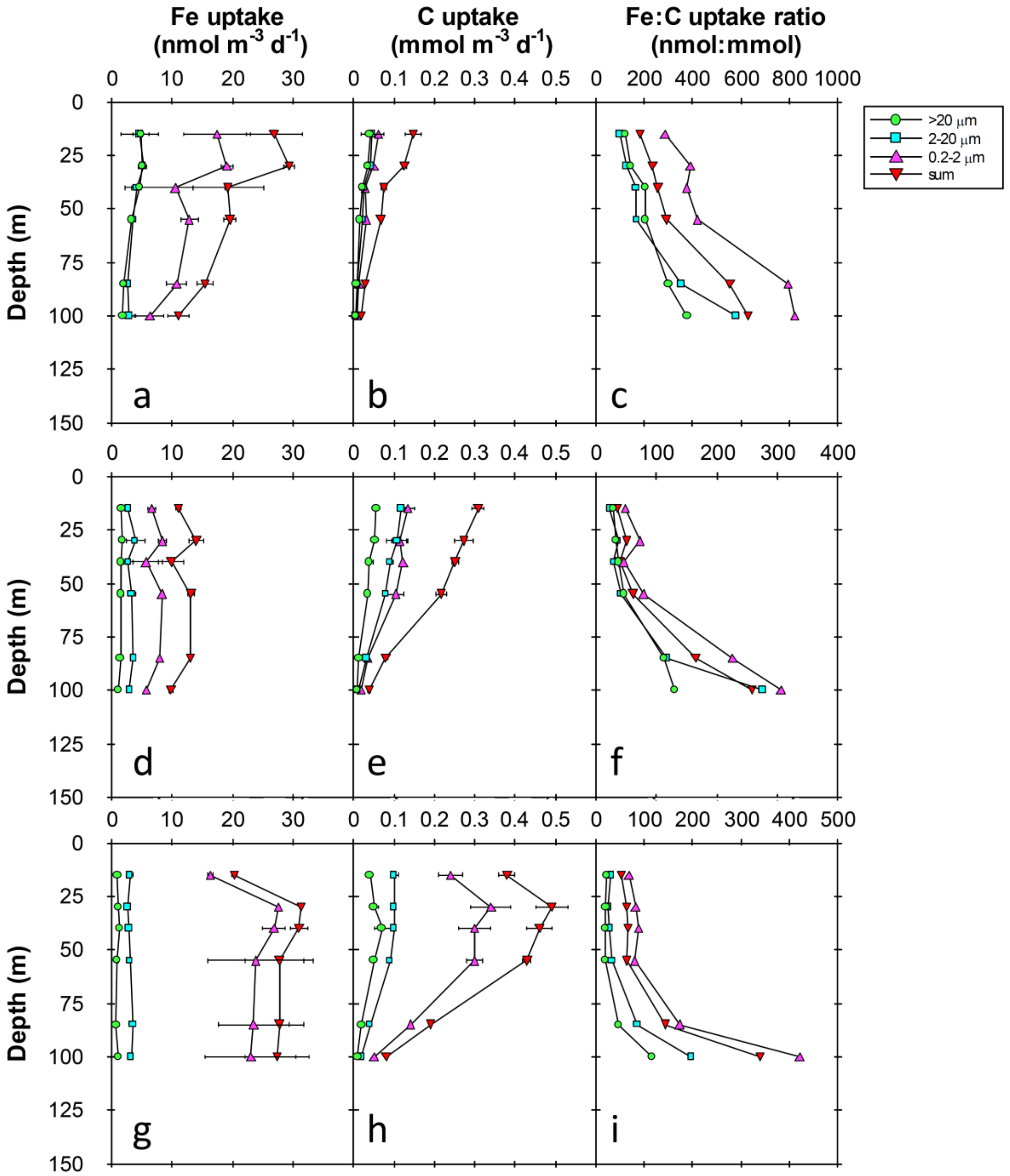

Supplementary Figure 4. Intercellular iron and carbon uptake rates. Size fractionated profiles of Fe (**a, d, g**), and carbon (**b, e, h**) uptake and the Fe:C uptake ratio (**c, f, i**) versus depth for the Cold Core eddy station (**a-c**), the Subantarctic zone station (**d-f**) and the Southern Ocean Time Series station (**g-i**). Error bars represent 1 s.d. for replicate measurements.

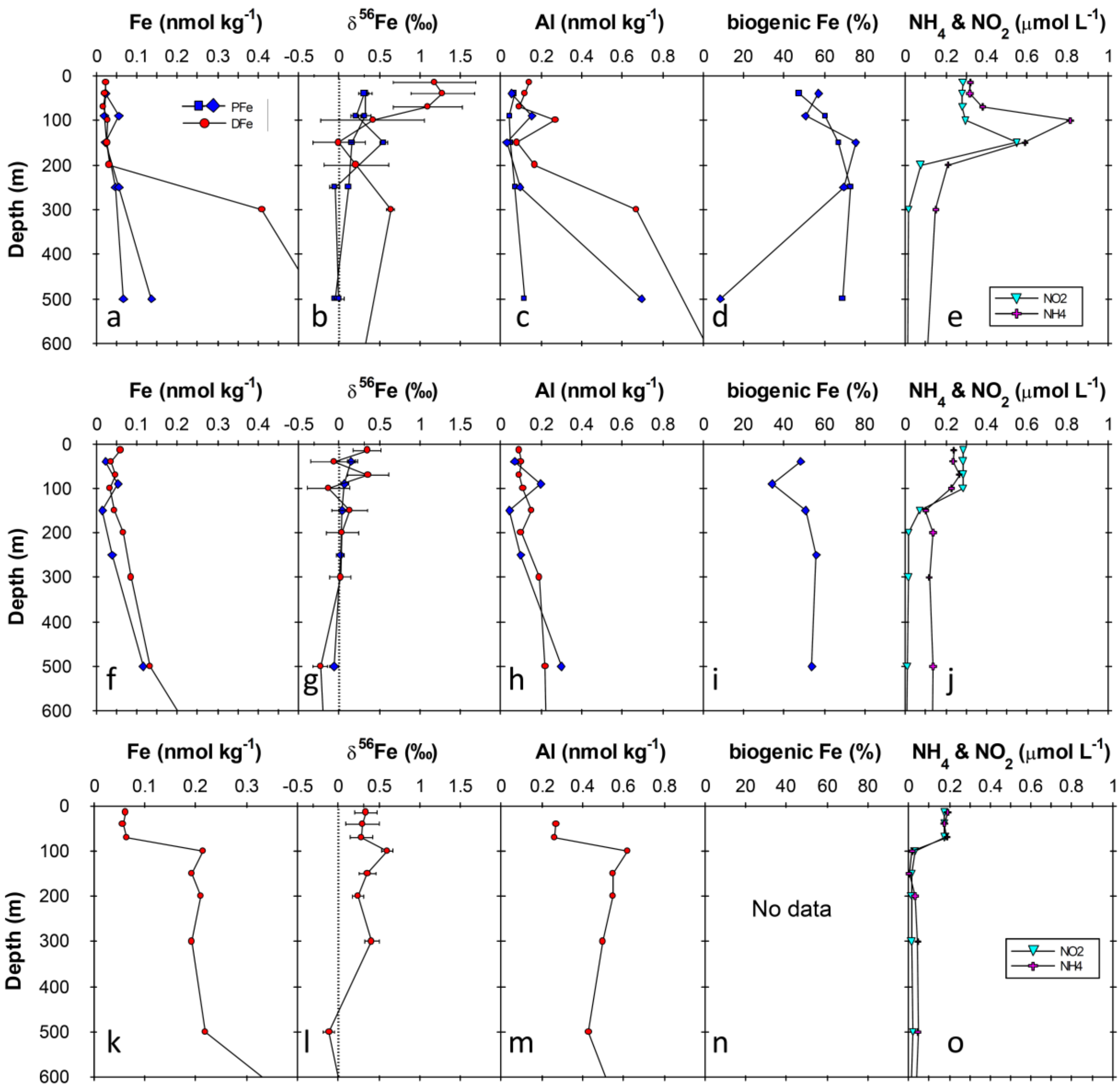

Supplementary Figure 5. Upper ocean metal and nutrient profiles. Depth profiles of dissolved (red symbols) and particulate (blue symbols) Fe concentration (**a, f, k**), and the Fe isotope composition of dissolved (red symbols) and particulate (blue symbols) Fe (**b, g, l**), dissolved (red symbols) and particulate (blue symbols) aluminium concentration (**c, h, m**), percentage biogenic Fe (**d, I, n**) and nitrite and ammonium concentration (**e, j, o**) for samples collected at the Cold Core eddy station (**a-e**), the Subantarctic zone station (**f-j**) and the Southern Ocean Time Series station (**k-o**). Biogenic Fe was calculated assuming that percentage biogenic Fe = $Fe_{tot.part.}$ – $Al_{tot.part.}$x $(Fe/Al)_{lithogenic}$ x 100. Where $Fe_{tot.part}$ is total particulate Fe concentration, $Al_{tot.part}$ is total particulate aluminium concentration and $(Fe/Al)_{lithogenic}$ is this Fe/aluminium ratio of lithogenic material. For this study, this was set to 0.18 based on the ratio from Frew et al. [21]. Error bars for isotope measurements represent 2 s.e.m.

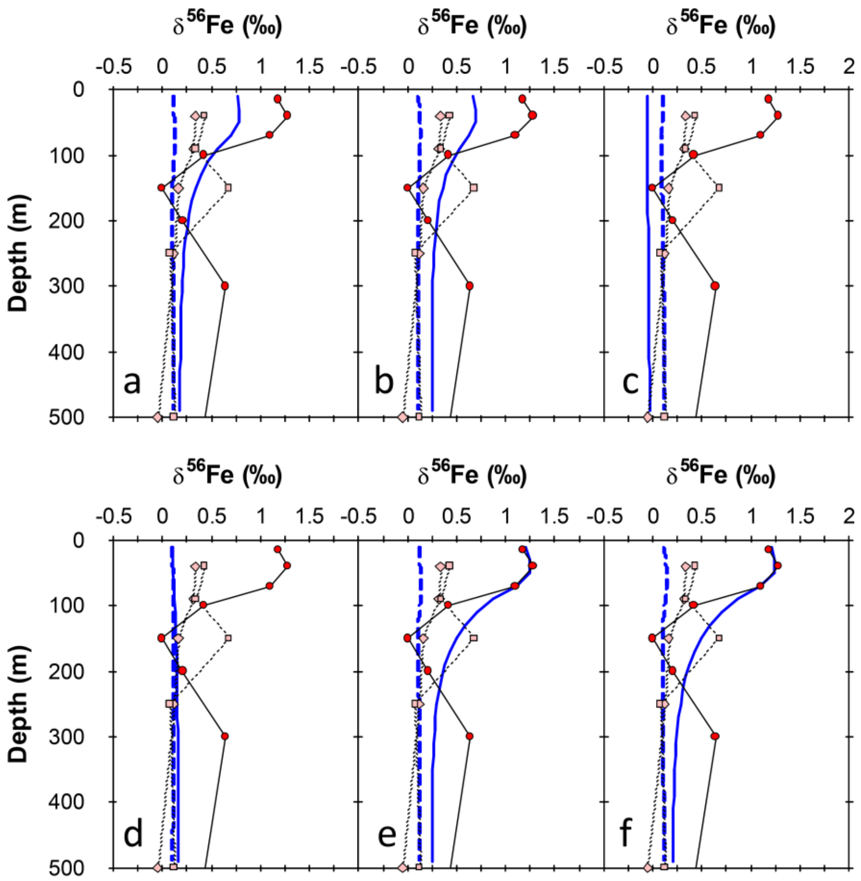

Supplementary Figure 6. One-D model output for processes involved in iron isotope fractionation. One-D model profiles (blue lines) for dFe and pFe versus depth for **a.** α_{uptake} = 0.9994 (ε = -0.6), **b.** $\alpha_{complexation}$ = 1.0006 (ε = +0.6), **c.** $\alpha_{regeneration}$ = 0.99985 (ε = +0.15) , **d.** $\alpha_{scavenging}$ = 0.9997 (ε = -0.6), **e.** α_{uptake} = 0.9994, $\alpha_{complexation}$ = 1.0006, $\alpha_{regeneration}$ = 0.99985, $\alpha_{scavenging}$ = 0.9997, and **f.** α_{uptake} = 0.999. Profiles of dFe and pFe isotope composition versus depth for the Cold Core Eddy are also presented in all panels.

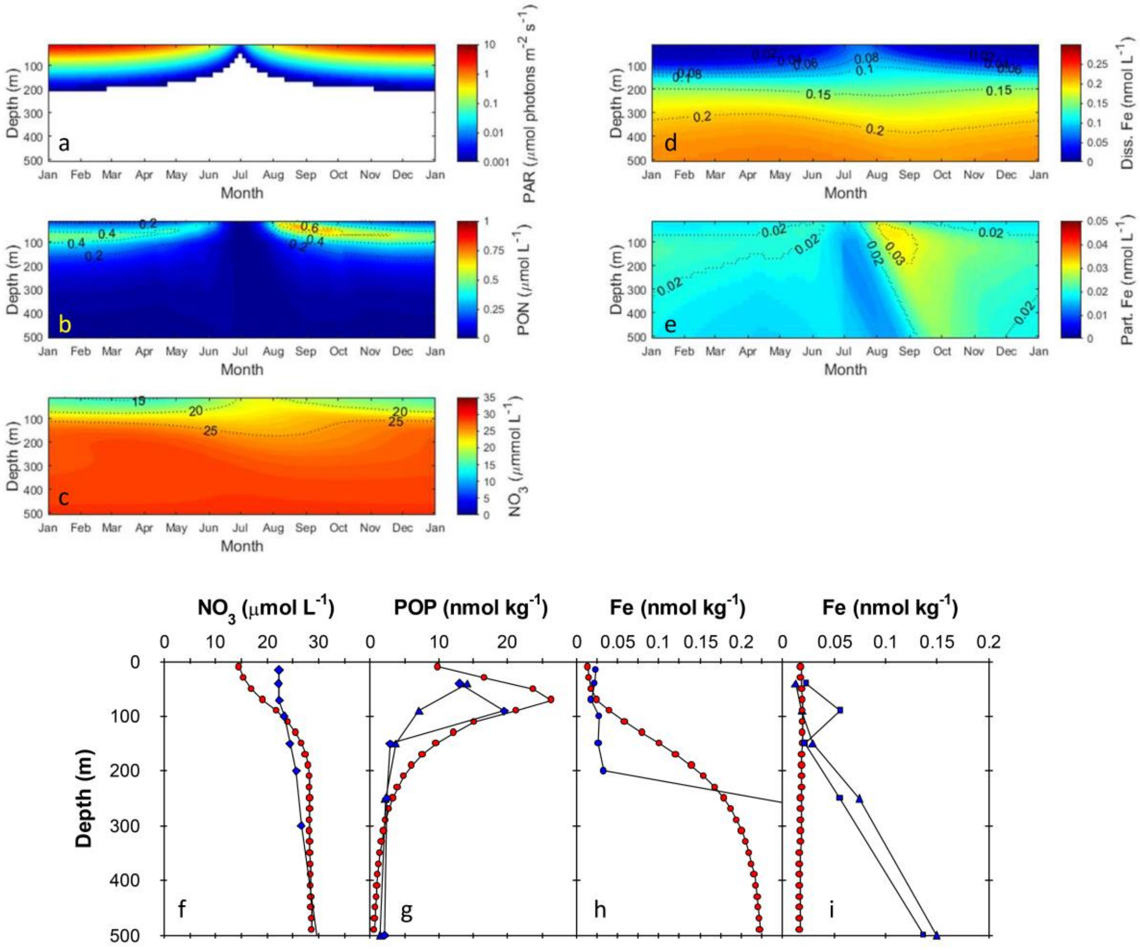

Supplementary Figure 7. One-D model output of seasonal nutrient and iron variations versus depth. Model output of seasonal variations in **a.** depth-resolved light penetration, **b.** phytoplankton concentration expressed as particulate organic nitrogen in the model, **c.** dissolved nitrate concentration, **d.** dissolved Fe concentration, **e.** particulate Fe concentration. Measured (Cold Core Eddy station data, blue symbols) and model values for 1 April (red symbols) versus depth for **f.** nitrate concentration, **g.** particulate phosphorous, **h.** dissolved Fe concentration and **i.** particulate Fe concentration.

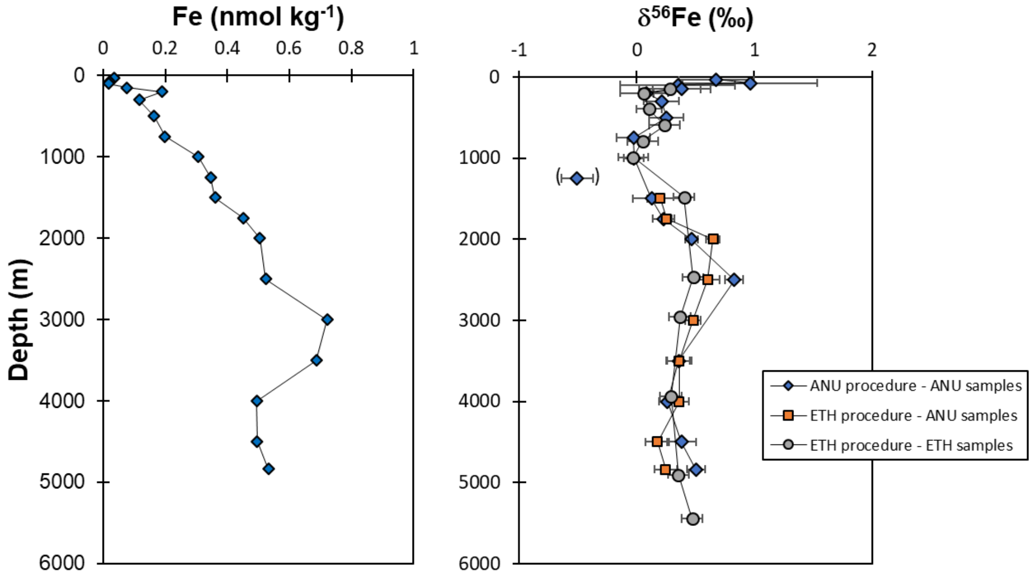

Supplementary Figure 8. Iron isotope results for samples collected on the GP13 and GP19 campaigns for a crossover station located at 32.5ºS, 170ºW in the South Pacific Ocean. **A.** The iron concentration results (left panel) are from the ANU group and have been published [22]. **B.** The iron isotope results are for samples collected and analysed by the ANU group, samples the ANU group shared with the ETH group and samples collected on GP19 and analysed by the ETH group [23,Conway in prep.]. One ANU sample analysis is highlighted as an outlier. The trends seen in the GP13 and GP19 profiles are consistent with each other and there is no systematic offset in the datasets. Error bars for isotope measurements represent 2 s.e.m..

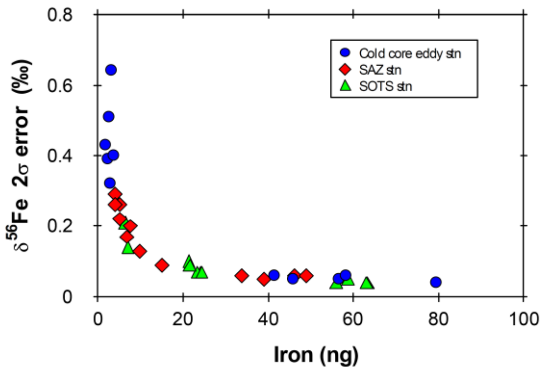

Supplementary Figure 9. Analytical error associated with Fe isotope values versus the quantity of Fe measured. The increase in analytical error is associated with instrumental noise (Johnson noise) when the amount of Fe analysed decreases. Data presented are for dissolved samples measured for the Cold Core eddy station, the Subantarctic zone station and the Southern Ocean Time Series station.

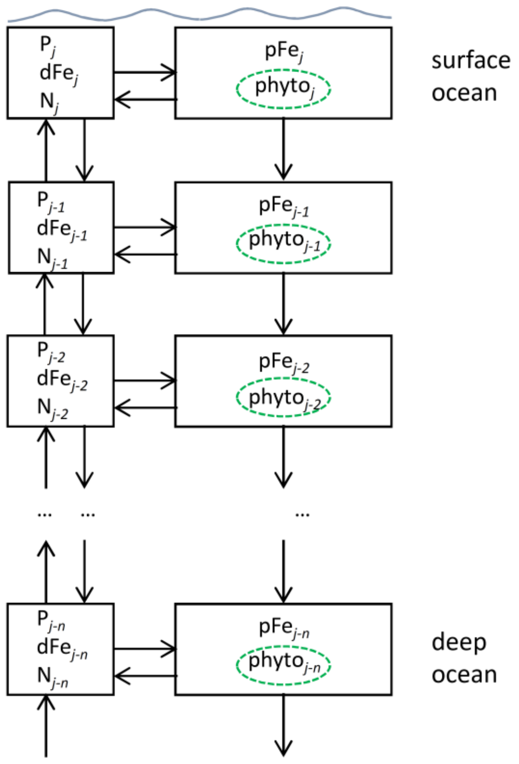

Supplementary Figure 10. Schematic representation of the 1D model configuration. Each box in the model was is 20 m deep and the model extends from 0-500 m. P_j, dFe_j, N_j, pFe_j and $phyto_j$, represent dissolved inorganic phosphate, iron, nitrate, particulate iron and phytoplankton, respectively for the j box.

Supplementary References

1 Schlosser, C. et al. Seasonal ITCZ migration dynamically controls the location of the (sub)tropical Atlantic biogeochemical divide. *Proc. Natl. Acad. Sci. USA* **111**, 1438-1442, (2014).
2 Martin, J. H., Knauer, G. A., Karl, D. M. & Broenkow, W. W. VERTEX- carbon cycling in the Northeast Pacific. *Deep-Sea Res. A.* **34**, 267-285 (1987).
3 Gledhill, M. & van den Berg, C. M. G. Determination of complexation of iron(III) with natural organic complexing ligands in seawater using cathodic stripping voltammetry. *Mar. Chem.* **47**, 41-54 (1994).
4 Rue, E. L. & Bruland, K. W. Complexation of iron(III) by natural organic ligands in the Central North Pacific as determined by a new competitive ligand equilibration/adsorptive cathodic stripping voltammetric method. *Mar. Chem.* **50**, 117-138 (1995).
5 Hudson, R. J. M., Covault, D. T. & Morel, F. M. M. Investigations of Iron Coordination and Redox Reactions in Seawater Using Fe-59 Radiometry and Ion-Pair Solvent-Extraction of Amphiphilic Iron Complexes. *Mar. Chem.* **38**, 209-235 (1992).
6 Johnson, K. S., Gordon, R. M. & Coale, K. H. What controls dissolved iron concentrations in the world ocean? *Mar. Chem.* **57**, 137-161 (1997).
7 Tagliabue, A. et al. Surface-water iron supplies in the Southern Ocean sustained by deep winter mixing. *Nat. Geosci.* **7**, 314-320, doi:10.1038/ngeo2101 (2014).
8 Dideriksen, K., Baker, J. A. & Stipp, S. L. S. Fe isotope fractionation between inorganic aqueous Fe(III) and a Fe siderophore complex. *Mineral. Mag.* **72**, 313-316 (2008).
9 Majestic, B. J., Anbar, A. D. & Herckes, P. Stable Isotopes as a Tool to Apportion Atmospheric Iron. *Environ. Sci. Technol.* **43**, 4327-4333 (2009).
10 Waeles, M., Baker, A. R., Jickells, T. & Hoogewerff, J. Global dust teleconnections: aerosol iron solubility and stable isotope composition. *Environ. Chem.* **4**, 233-237 (2007).
11 John, S. G. & Adkins, J. The vertical distribution of iron stable isotopes in the North Atlantic near Bermuda. *Global Biogeochem. Cycles* **26**, GB2034 (2012).
12 Albani, S. et al. Improved dust representation in the Community Atmosphere Model. *J. Adv. Model. Earth Syst.* **6**, 541-570 (2014).
13 Strzepek, R. F., Maldonado, M. T., Hunter, K. A., Frew, R. D. & Boyd, P. W. Adaptive strategies by Southern Ocean phytoplankton to lessen iron limitation: Uptake of organically complexed iron and reduced cellular iron requirements. *Limnol. Oceanogr.* **56**, 1983-2002 (2011).
14 Raimbault, P. & Garcia, N. Evidence for efficient regenerated production and dinitrogen fixation in nitrogen-deficient waters of the South Pacific Ocean: impact on new and export production estimates. *Biogeosciences* **5**, 323-338 (2008).
15 Harrison, W. G., Harris, L. R. & Irwin, B. D. The kinetics of nitrogen utilization in the oceanic mixed layer: Nitrate and ammonium interactions at nanomolar concentrations. *Limnol. Oceanogr.* **41**, 16-32 (1996).
16 Ammerman, J. W., Hood, R. R., Case, D. A. & Cotner, J. B. Phosphorus deficiency in the Atlantic: An emerging paradigm in oceanography. *Eos, Transactions American Geophysical Union* **84**, 165-170 (2003).
17 Sunda, W. G., Swift, D. G. & Huntsman, S. A. Low Iron Requirement for Growth in Oceanic Phytoplankton. *Nature* **351**, 55-57 (1991).
18 Moore, L. R., Goericke, R. & Chisholm, S. W. Comparative Physiology of Synechococcus and Prochlorococcus - Influence of Light and Temperature on Growth, Pigments, Fluorescence and Absorptive Properties. *Mar. Ecol. Prog. Ser.* **116**, 259-275 (1995).

19 Raimbault, P., Garcia, N. & Cerutti, F. Distribution of inorganic and organic nutrients in the South Pacific Ocean - evidence for long-term accumulation of organic matter in nitrogen-depleted waters. *Biogeosciences* **5**, 281-298, doi:10.5194/bg-5-281-2008 (2008).

20 Moore, J. K. & Braucher, O. Sedimentary and mineral dust sources of dissolved iron to the World Ocean. *Biogeosciences* **5**, 631-656 (2008).

21 Frew, R. D. et al. Particulate iron dynamics during FeCycle in subantarctic waters southeast of New Zealand. *Global Biogeochem. Cycles* **20** GB1S93 (2006).

22 Ellwood, M. J. et al. Insights Into the Biogeochemical Cycling of Iron, Nitrate, and Phosphate Across a 5,300 km South Pacific Zonal Section (153°E–150°W). *Global Biogeochem. Cycles*, 32, 187-207 (2018).

23 Conway, T.M., Sieber, M., Ellwood, M.J., Takano, S., Sohrin, Y. and Vance, D. CT31A-03: The competing influence of local cycling, regional sources and Southern Ocean processes in influencing Fe isotope cycling at lower latitudes in the oceans, Ocean Sciences Meeting, Portland, Oregon (2018).
