# Supplementary material for: Distinct iron cycling in a Southern Ocean eddy
Source: Nat Commun. 2020 Feb 11;11:825. doi: 10.1038/s41467-020-14464-0 (PMC7012851; doi:10.1038/s41467-020-14464-0)
Supplement: Supplementary file 4 — Description of Additional Supplementary Files [file 41467_2020_14464_MOESM4_ESM.docx]

Description of supplementary information

Title: Supplementary movie

Description: Evolution of the core cold eddy. Eight-day averaged satellite images of a. chlorophyll a and b. sea surface temperature for the period between 5 January 2016 to 20 May 2016. Also shown are the locations of Cold Core eddy station (CCE), the Subantarctic zone station (SAZ) and the Southern Ocean Time Series station (SOTS) and the solid black line represents the Triaxus tow. Eight-day averaged satellite images of The contour lines in each panel are for surface sea surface temperature. The 8-day averaged chlorophyll a satellite data were extracted from https://coastwatch.pfeg.noaa.gov/erddap/griddap/ while the 8-day averaged sea surface temperature data were obtained from the Australian Ocean Data Network (https://portal.aodn.org.au/) portal. Black contours lines are for sea surface temperature on both panels. White areas indicated no image data.
